# Serum Exosomes and Their miRNA Load—A Potential Biomarker of Lung Cancer

**DOI:** 10.3390/cancers13061373

**Published:** 2021-03-18

**Authors:** Mateusz Smolarz, Piotr Widlak

**Affiliations:** Maria Skłodowska-Curie National Research Institute of Oncology, Gliwice Branch, 44-101 Gliwice, Poland; mateusz.smolarz@io.gliwice.pl

**Keywords:** biomarkers, exosome, extracellular vesicles, lung cancer, miRNA, plasma, serum

## Abstract

**Simple Summary:**

Exosomes are an emerging source of cancer biomarkers. Molecular components of serum-derived exosomes have been addressed in several reports in the context of biomarkers for early detection of lung cancer. However, despite the promising results of pilot studies, the clinical applicability of such biomarkers has not been validated yet. In this review, the diagnostic potential of miRNA content of serum-derived exosomes is presented. Moreover, potential target genes and signaling pathways affected by miRNA present in lung cancer signatures are discussed.

**Abstract:**

Early detection of lung cancer in screening programs is a rational way to reduce mortality associated with this malignancy. Low-dose computed tomography, a diagnostic tool used in lung cancer screening, generates a relatively large number of false-positive results, and its complementation with molecular biomarkers would greatly improve the effectiveness of such programs. Several biomarkers of lung cancer based on different components of blood, including miRNA signatures, were proposed. However, only a few of them have been positively validated in the context of early cancer detection yet, which imposes a constant need for new biomarker candidates. An emerging source of cancer biomarkers are exosomes and other types of extracellular vesicles circulating in body fluids. Hence, different molecular components of serum/plasma-derived exosomes were tested and showed different levels in lung cancer patients and healthy individuals. Several studies focused on the miRNA component of these vesicles. Proposed signatures of exosome miRNA had promising diagnostic value, though none of them have yet been clinically validated. These signatures involved a few dozen miRNA species overall, including a few species that recurred in different signatures. It is worth noting that all these miRNA species have cancer-related functions and have been associated with lung cancer progression. Moreover, a few of them, including known oncomirs miR-17, miR-19, miR-21, and miR-221, appeared in multiple miRNA signatures of lung cancer based on both the whole serum/plasma and serum/plasma-derived exosomes.

## 1. Introduction

Lung cancer is among the major cancer-related public health problem responsible for about a quarter of cancer-related deaths worldwide. Overall, the lung cancer five-year survival rate (below 20%) is much lower than other leading cancer sites, such as colorectal (about 65%), breast (about 90%), and prostate (about 95%). Though the risk and incidence of lung cancer are slightly higher among men, this malignancy is becoming the major cause of cancer-related death also in women. The majority of lung cancer cases are diagnosed at advanced stages and have unfavorable prognoses (the average five-year survival of about 10–15%). However, in the case of the disease detected at the early stages, the prognosis is much better (the average five-year survival varies between 65 and 85%). Thus, in addition to primary prevention (i.e., tobacco smoking control), screening for early detection was proposed as a promising strategy to reduce lung cancer mortality [1,2]. Several screening tools have been investigated during the past decades, but only one, the low-dose computed tomography (LD-CT), has found an application in clinical practice. Originally, the results of the National Lung Screening Trial (NLST) showed that compared to chest X-ray examination, the LD-CT screening was associated with over 20% reduction of lung cancer-specific mortality in a high-risk group of subjects defined by their smoking status and age [3]. The potential of LD-CT screening programs to reduce lung cancer mortality was further confirmed by other studies [2], including the Dutch–Belgian NELSON trial [4] and the Danish Lung Cancer Screening Trial (DLCST) [5]. It is estimated that the use of LD-CT allows for earlier detection of lung cancers in about 12,000 people a year, which is about 8% of deaths annually due to this disease. It is worth noting, however, that LD-CT allows detecting abnormalities in 20–40% of people undergoing this examination, but as much as 95% of results could be false-positive [6]. Hence, due to the low specificity of LD-CT (positive predictive value of only 3.8% in the NLST), the vast majority of patients with screen-detected chest abnormalities are subjected to further expensive and potentially harmful diagnostic procedures, such as transthoracic or bronchoscopic biopsy or surgery. It is estimated that about 75% of patients unnecessarily underwent diagnostic workup, including 25% subjected to invasive procedures [7]. Hence, there is an urgent need for clinical and molecular tests supporting CT-based screening for the detection of lung cancer to reduce “over-diagnosis” and decrease the costs. Such test(s) could either pre-select individuals for LD-CT examination or discriminate between benign and malignant chest abnormalities detected by LD-CT [8,9].

Potential biomarkers for early lung cancer can be found in various biological fluids; however, blood is the richest and most readily available source [10,11]. Candidates for such biomarkers include serum proteins, free nucleic acids, and metabolites [11,12]. Several works reported serum/plasma proteins, which levels are associated with the risk of lung cancer [13]. Another candidate for the biomarker of lung cancer is circulating free DNA (cfDNA) [14] and circulating tumor cells (CTC) [15]. More recently, serum metabolites and lipids have emerged as another class of potential biomarkers in lung cancer [16,17]. Several other review papers could be suggested that cover this well-researched field [11,12,13,18,19,20,21]. However, though numerous biomarker candidates have been proposed only a few of them have been positively validated in the proper clinical settings. The main reason was the lack of sensitivity and analytical reproducibility, which in turn led to the elimination of potential candidates from further stages of biomarker testing [9,12]. Moreover, none of the tested biomarkers increased the actual number of detected early lung cancer cases yet [18,20,22]. Currently, only two molecular tests are used in clinical practice to help in the diagnosis of indeterminate pulmonary nodules detected by CT. One of them is the autoantigen-based EarlyCDT-Lung test, which enables the classification of indeterminate nodules with a positive predictive value (PPV) >70% [23]. Another test is the XL2 test, which combines the clinical probability of cancer score with the level of two plasma proteins: LG3BP and C163A [24]. Hence, the identification of the reliable molecular biomarker that could be used for the early detection of lung cancer remains a timely and vital issue. 

The purpose of this literature review is to summarize current data on the emerging biomarker of early lung cancer-circulating serum exosomes and their microRNA cargo.

## 2. Micro RNA Signatures of Lung Cancer

In the search for a lung cancer biomarker, there were numerous studies focused on microRNAs (miRNAs). It is a class of small endogenous non-coding RNAs of 18–24 nucleotides responsible for the regulation of target genes. More than 2500 mature miRNAs have been described in humans yet [25,26,27]. miRNA is transcribed in the cell nucleus with the participation of RNA polymerase II resulting in pri-miRNA, which is processed by the Drosh/DGCR8 enzyme complex to precursor miRNA (pre-miRNA). The resulting pre-miRNA is transported from the nucleus to the cytoplasm involving Exportin-5, where it is processed by Dicer nuclease to form miRNA duplexes or mature miRNA. Usually, a less-thermostable 5’-terminus strand is packed to the protein complex (RISC), whose main component is a protein from the Argonaut family (AGO), while the second strand is degraded. The RISC complex then recognizes the target mRNA and binds at the 3’UTR position: mRNA degradation occurs in the case of perfect miRNA/mRNA matching, while translation repression in the case of incomplete alignment. Thus, by silencing target mRNAs, miRNAs affect many critical cellular processes such as cell proliferation, apoptosis, differentiation, and metabolism [27,28]. 

The composition of miRNA component of tissues (so-called miR-ome) could be affected by different pathological conditions; hence, the diagnostic and prognostic values of miRNA signatures have been addressed in many studies [29,30,31,32,33,34]. miRNA is resistant to RNase digestion, boiling, extended storage, extreme pH, and multiple freezing and thawing cycles [35]. Moreover, miRNA is considered to be more stable than other classes of RNA in blood and other biofluids. However, it should be noted that during the analysis of free circulating miRNA in human blood, miRNA molecules released by cancer cells and other classes of “normal” cells (platelets, red blood cells, and endothelial cells) are co-purified and co-analyzed [36]. Nevertheless, miRNA circulating in the blood and present in the isolated serum (i.e., the liquid fraction of blood remaining after removal of the clot followed coagulation) or plasma (i.e., the liquid fraction of blood remaining after removal of cell components without coagulation), is an emerging source of disease biomarkers including lung cancer. 

Several studies addressed circulating miRNA as potential molecular signatures to be used for the diagnosis of lung cancer. Numerous papers have been published since 2011 that described signatures of serum/plasma miRNA, which enabled the differentiation between lung cancer patients and healthy individuals. Some of these reports described single miRNA, yet most of them proposed multi-component panels up to 24 plasma miRNAs [37] or 34 serum miRNAs [38]. Examples of such studies are listed in Table 1. Proposed lung cancer signatures involved about 100 miRNA species overall, which (according to our literature review) included 39 miRNA species that recurred in more than one signature. However, only four miRNA species were included in more than five signatures, namely, miR-21 (11 signatures), miR-148b (8 signatures), miR-126, and miR-486–5p (seven signatures). Hence, the overlap among different signatures was relatively low, which putatively reflected different clinical characteristics of lung cancer patients and their ethnic/genetic backgrounds as well as different analytical approaches used in different studies. Nevertheless, we analyzed a subset of 39 miRNA species that appeared in multiple lung cancer signatures in the search for their target genes and associated biological functions; the bioinformatics tool miRSystem (version 20160513) was used [39]. Among the biological processes associated with this subset of miRNAs and statistically overrepresented, several pathways were involved in cancer development, including the MAPK signaling, FGFR signaling, transport of glucose, apoptosis, and antigen processing/presentation. This subset included several known “oncomirs”, exemplified by miR-21, which will be discussed in detail below. Furthermore, among the genes hypothetically targeted by the highest number of miRs from this subset were a few genes with putative cancer-related functions, exemplified by *IFI30*, *PLA2G10*, *FGF6*, *ZBTB16*, and *CORO1A*. *IFI30* encodes a lysosomal thiol reductase involved in the processing of MHC class II-restricted antigen, which was reported in the development of melanoma [40]. *PLA2G10* encodes a phospholipase A2 family member involved in the production of inflammatory lipid mediators (e.g., prostaglandins), which was reported in the progression of breast cancer [41]. *FGF6* encodes a fibroblast growth factor (FGF) family member involved in tumor growth [42]. *ZBTB16* encodes a Krueppel C2H2 zinc finger family member involved in the regulation of cell cycle, apoptosis, and the AKT/Foxo3a pathway [43]. *CORO1A* encodes a WD-repeat protein family member involved in the cell cycle progression, apoptosis, and signal transduction [44]. Hence, cancer-related functions of miRNA species present in the proposed lung cancer signatures provide additional validation of their putative diagnostic importance.

In conclusion, circulating miRNA appears a forward-looking diagnostic tool in the detection of lung cancer. Proposed signatures revealed promising sensitivity and specificity, which usually reached 80–90%. Still, their actual diagnostic reproducibility requires further validation and clinical testing [25,35,45,46,47]. Further, none of the proposed miRNA signatures have yet been conclusively validated in the prospective clinical studies. Nevertheless, three registered clinical trials are currently ongoing that include validation of the serum/plasma miRNA signatures of early lung cancer. The BIOMILD study (NCT02247453) sponsored by the Fondazione IRCCS Istituto Nazionale dei Tumori (Milano) is aimed at the validation of the Plasma miR Signature Classifier [37]. The COSMOS study (NCT01248806) sponsored by the European Institute of Oncology involves validation of the miR-Test [48] in the context of lung cancer screening. Moreover, a smaller study sponsored by Hummingbird Diagnostics (NCT03452514) is aimed at the validation of the commercial HMBDx microRNA Test in a group of participants of the LD-CT lung cancer screening. However, all these clinical trials are still running, and no conclusions are available yet (the planned completion date of these studies is 2021).

## 3. Exosomes, an Emerging Type of Liquid Biopsy

**Figure 1:**
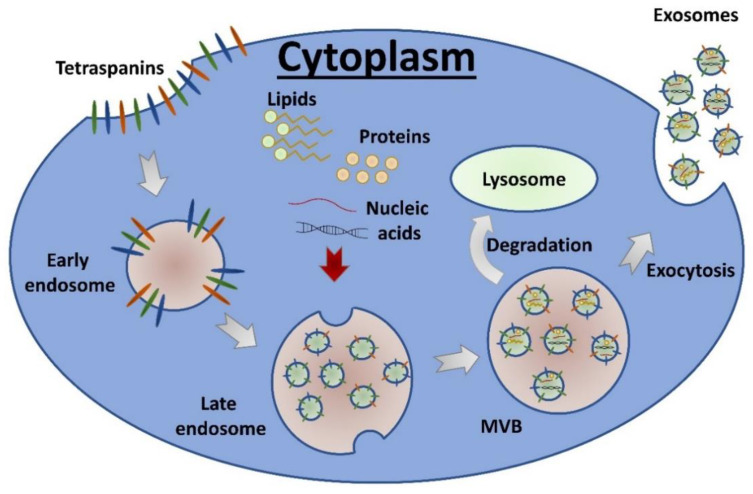
Biogenesis of exosomes.

Exosomes are membrane-enclosed nanovesicles (30–150 nm) of endosomal origin. Exosomes arise as a result of the concavity of the plasma membrane inward, resulting in the formation of an early endosome. The early endosome matures into the late endosome, which then transforms into a multivesicular body (MVB) that could attach to the plasma membrane from inside and release exosomes into the extracellular space [64,65] (Figure 1). Exosomes can be detected in various biological fluids such as urine, cerebrospinal fluid, saliva, blood, and its derivatives (serum and plasma). Exosomes are secreted by all types of cells, either non-tumorigenic and cancerous. These vesicles are enclosed by a double film of symmetrically distributed lipids containing several tetraspanins and other membrane proteins involved in the formation of MVB (CD9, CD63, CD81, TSG101, and Alix). However, the full set of proteins present in the exosome cargo (involving thousands of different cellular proteins) is variable and reflects the current phenotype of the parent cell. Except for proteins and lipids, exosomes also contain different classes of nucleic acids (single-stranded RNA, long non-coding RNA, and microRNA) and metabolites, whose composition is also regulated by the state of the cell [64,66,67].

In general, exosomes are involved in many aspects of cell-to-cell communication working in both paracrine and endocrine modes. In the case of exosomes from “normal” (non-tumorogenic) cells, their role in immunity, coagulation, angiogenesis, spermatogenesis, and various physiological processes in the central nervous system has been confirmed. In the case of tumor-derived exosomes (TEX), several lines of evidence indicate their association with immunomodulation, pre-metastatic niche formation, tumor growth, resistance to the treatment, and drug removal from cells [68]. TEX are signal mediators and promote disease development by participating in processes such as angiogenesis, metastasis, and many others [66,68,69,70]. TEX are released into the bloodstream so they can reach distant organs and modify the phenotype of many different cell types. This ability of TEX depends on their bioactive cargo, which differs from the content of exosomes released by “normal” cells and corresponds to the malignant phenotype of cancer cells [71]. Several review papers focused on the functional role of TEX have already been published, including a few recent ones [68,70,72,73]

Exosomes released by lung cancer cells were reported to be involved in tumor promotion, immunomodulation, and remodeling of the tumor microenvironment, also in the context of metastatic niche [66,69]. TEX secreted by lung cancer cells contain several proteins involved in tumor development, including CD91, Galectin-9, LRG1, EGFR, and Wnt5b [53,70,73,74,75,76]. Several studies also addressed the functional importance of non-coding RNA present in TEX released by lung cancer cells. For example, miR-103a present in TEX directly affected the polarization of macrophages by reducing PTEN protein expression, which in turn led to the accumulation of tumor-promoting factors such as IL10, CCL2, and VEGF-A [70,77]. Moreover, miR-21 present in TEX promoted tumor growth by increasing the permeability of blood vessels and the accumulation of hypoxia-induced factor-1α (HIF-1α) under both normoxic and hypoxic conditions [78]. Other miRNAs present in TEX secreted by lung cancer cells (e.g., miR-9, miR-126, miR-122, and miR-210) could also participate in the process of angiogenesis of neoplastic blood vessels [73,74,79,80,81,82]. Long non-coding RNAs (lncRNAs) are another group of nucleic acids present in TEX secreted from lung cancer cells. It has been reported that several such lncRNAs (MALAT1, AK126698, SCAL1, and HOTAIR) are associated with the anti-apoptotic activity, resistance to cisplatin, protection of cells against oxidative stress, and increased migration proliferation and invasiveness [74,79,83,84]. 

The molecular composition of TEX reflects that of parental cancer cells. Therefore, TEX present in blood and other biofluids are an emerging type of liquid biopsy, considered a gold mine of potential cancer markers [26,72,85,86,87]. It should be emphasized, however, that exosomes represent only a subset of the heterogeneous group of extracellular vesicles (EV) that also include microvesicles (also known as ectosomes; 250–1000 nm) and apoptotic bodies (>1000 nm) formed by outward budding (“blebbing”) of the plasma membrane. The term “exosomes” should be reserved for vesicles of endosomal origin that form via MVB. However, due to the limitations of current methods used for the isolation of EV the adequate discrimination between various EV subsets is not feasible. Therefore, to avoid possible misconceptions, a simplified nomenclature has been recently proposed that distinguishes small EV (i.e., <200 nm) and medium/large EV (>200 nm). A class of small EV (sEV) consists mostly of exosomes, yet other types of EV, e.g., small microvesicles, could also copurify with this fraction [88]; in this review, the terms “exosome” and “sEV” are used interchangeably for simplicity. Moreover, sEV present in blood and other biofluids represent a complex mixture of vesicles released by different types of cells. It is estimated that TEX represent about 20–60% of sEV present in the plasma of cancer patients while the remaining exosomes and other sEV present in this specimen are released by “normal” non-cancerous types of cells (e.g., platelets, immune cells, and endothelial cells) [89]. However, due to current limitations of methods allowing purification of specific TEX from body fluids [90], the mixture of different sEV that could be isolated from serum or plasma remains a feasible material in the search of cancer markers. Nevertheless, even such heterogeneous material is a promising source of biomarkers for the detection of lung cancer, which is discussed below.

## 4. Serum Exosomes as Potential Lung Cancer Biomarkers

Exosomes are secreted by various cells. However, the concentration of exosomes is much higher in the blood of cancer patients, including lung cancer, compared to healthy individuals. Recent reports indicate that the concentration of vesicles in the blood of cancer patients may reach 10^9^ vesicles/mL of blood [71]. The above observations have been confirmed in many types of cancers, including prostate cancer, ovarian cancer, breast cancer, pancreatic ductal adenocarcinoma, hepatocellular carcinoma, and breast cancer [91,92,93,94,95]. Increased levels of vesicles in the blood of cancer patients correlate with a worse prognosis. The molecular cargo of exosomes is the primary source of cancer biomarkers. However, apart from a different molecular cargo, TEX may have a different morphology than exosomes secreted by “normal” cells. Exosomes isolated from the serum of patients diagnosed with pancreatic cancer had a significantly smaller size compared to exosomes isolated from healthy people [91]. Similar observations were made with the use of atomic force microscopy in the case of exosomes present in patients with oral cancer [96]. Hence, the number, composition, and morphology of exosomes can be an important diagnostic cancer biomarker, though no specific data regarding lung cancer patients is available yet.

Different molecular components of exosomes existing in body fluids (serum, plasma, and saliva) of patients with lung cancer have been tested in the search for a biomarker of this malignancy [85,97,98,99,100,101,102]. Identified biomarker candidates include different classes of molecules-nucleic acids, proteins, and metabolites. Results of these studies (except for exosome miRNA discussed in the subsequent paragraph) are listed in Table 2. A few signatures of lung cancer have been proposed based on proteins present in serum/plasma-derived exosomes [86,103,104,105,106,107]. Moreover, several studies have proposed long non-coding RNAs and circular RNAs present in serum-derived exosomes as lung cancer biomarkers [84,108,109,110,111]. Furthermore, different levels of several phospholipids (phosphatidylcholines and sphingomyelins), triglycerides, and cholesterol esters present in the exosome membrane have been observed in plasma-derived exosomes in lung cancer patients and healthy controls [112]. Different diagnostic performance of proposed signatures was reported (Area Under the ROC Curve, AUC, was in the range 0.70 to 0.90), yet the observed difference could be attributed to differences in the statistical methodology. Nevertheless, though some of these biomarker candidates are promising, their actual diagnostic performance has not yet been validated in the proper clinical settings.

## 5. Exosome miRNA as a Biomarker of Lung Cancer

The miRNA content of serum/plasma-derived exosomes is another promising source of lung cancer biomarkers addressed in several papers. Two analytical methods of miRNA detection dominate in these studies—quantitative PCR and next-generation sequencing. However, many different approaches were applied to isolate and characterize sEV from serum or plasma; hence, different classes of vesicles could be studied in different reports. The representative papers are summarized in Table 3. Some of these studies tested the diagnostic performance of miRNA signatures, which resulted in AUC values that ranged between 0.71 and 0.98. However, none of these signatures have yet been validated in an independent study. Furthermore, none of them have been studied in the context of lung cancer screening. Analyzed groups had different sizes and represented different clinical characteristics and ethnic/genetic backgrounds. Therefore, different miRNA signatures of serum/plasma exosomes proposed to discriminate lung cancer patients from healthy controls should be compared with caution.

**Figure 2:**
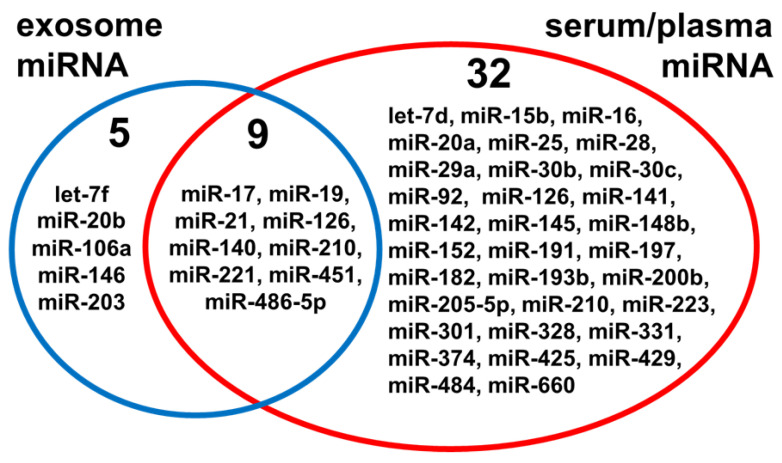
MicroRNA species present in lung cancer signatures. Showed are components present in at least 2 signatures identified in either whole serum/plasma or serum/plasma-derived exosomes (small extracellular vesicles).

According to current literature research, proposed lung cancer exosome signatures involved above 60 miRNA species overall, and 14 miRNA species appeared in more than one signature. This included miR-21 (seven signatures), miR-221 (three signatures), and miR-486-5p (three signatures). Figure 2 illustrates miRNA species present in lung cancer signatures, detected in either whole serum/plasma or serum/plasma-derived exosomes, which were included in more than one signature. There were nine miRNA species, namely, miR-17, miR-19, miR-21, miR-221, miR-451, miR-486-5p, miR-126, miR-140, and miR-210, which appeared in both whole serum/plasma and exosome-based signatures. Functions associated with this interesting subset of miRNAs are discussed below.

Shared components of the whole serum/plasma-based and exosome-based lung cancer signatures contain several oncomirs, i.e., miRNAs with known cancer-related functions. These include miR-17 and miR-19 belonging to the miR-17-92 cluster, which is regulated by MYC. The miR-17-92 cluster is a unique oncomir due to the polycistronic miRNA transcript, which allows obtaining six individual miRNAs involved in many cancer-associated processes: miR-17, miR-18a, miR-19a, miR-20a, miR-19b-1, and miR-92a-1 [137]. A high level of miR-17 and miR-19 induces cell proliferation, while the deletion is lethal (it causes lung and lymphoid cell developmental defects) [138]. miR-17 suppresses the expression of the E2F1 transcription factor, shifting the cellular balance in favor of increased proliferation. In lung cancer, overexpression of miR-17 and miR-19 affects the expression of *HIF1A*, *PTEN*, *BCL2L11*, *CDKNA*, and *TSP1*, enhancing tumor growth by increasing the permeability of blood vessels, inducing hypoxia, increasing proliferation, inhibiting apoptosis, and stimulating tumor cell migration [139,140]. miR-21 is another oncomir frequently overexpressed in cancer cells, one of the first miRNAs identified in mammals. Among the targets of miR-21 are tumor suppressor genes such as *PTEN*, *RHOB*, and *TP63*. Further, miR-21 blocks AKT and MAPK signaling pathways via inhibition of several phosphatases. As a result of miR-21 overexpression, the action of tumor suppressors is blocked, causing the development of many cancers such as lung, ovarian, breast, brain, and many others [141]. In lung cancer, overexpression of miR-21 is associated with increased cell proliferation, angiogenesis, cell invasion, and metastasis, as well as chemo- and radioresistance [142]. The inhibition of miR-21 resulted in the induction of apoptosis (due to inhibiting the PI3K/Akt/NF-KB signaling pathway and increased caspase activity) as well as impeded the migration and invasiveness of NSCLC cells [143]. miR-21 is involved in modulating the tumor microenvironment by targeting *PTEN* in the stromal compartment, which is mediated by miR-21-containing TEX [144]. Another oncogenic miRNA found in TEX is miR-221. miR-221 inhibits p27 tumor suppressor, which causes the transition from G1 to S phase and acceleration of cell division [145]. Among miR-221 targets is also *CD117*, a known proto-oncogene that regulates cell survival, migration, and differentiation. Overexpression of miR-221 induces proliferation and migration of tumor cells as well as tumor angiogenesis via the Wnt/β-catenin signaling pathway and has been shown to promote the chemoresistance of lung cancer cells by activating the PTEN/Akt pathway [146]. miR-210 is also an important factor in the development of lung cancer, whose level increases in NSCLS tissues and is associated with a worse prognosis [147]. The action of miR-210 involves the regulation of HIF-1, ATG7, LC3, and Beclin-1 [148].

Other miRNA observed in multiple lung cancer signatures are putative tumor suppressors. In lung cancer, a decreased level of miR-451 correlates with poor prognosis [149]. Functionally, decreased expression of miR-451 increases drug resistance and accelerates the epithelial-mesenchymal transition due to *MYC* overexpression, which is a miR-451 target [150]. Moreover, miR-451 targets several genes involved in the inflammation and stress response pathways that modulate the tumor microenvironment, including *PSMB8*, *NOS2*, and *CARF* [151]. Another component of exosome lung cancer signature is miR-126, which level was reduced in cancer patients. Overexpression of miR-126 inhibits cancer cell proliferation, colony formation, migration, invasion, induces cell cycle arrest, and apoptosis via targeting *ITGA6* gene [152]. Another characteristic component of serum-derived exosomes is miR-140 involved in carcinogenesis and tumor progression, which level is significantly lowered in tumors. Overexpression of miR-140 is associated with inhibition of proliferation, migration, and invasion of NSCLC cells via targeting of *ATP8A1* and *IGF1R* genes [153,154]. Another miRNA shared by serum and exosome-based signatures is miR-486-5p. This miRNA, one of the most abundant miRNAs in the peripheral blood, plays an important role in the development of many cancers. Overexpression of miR-486-5p increases cell proliferation by regulating the PTEN/PI3K/AKT pathway [155]. On the other hand, however, decreased levels of miRNA-486-5p in NSCLC tissues correlated with increased drug resistance and a worse prognosis [156]. Moreover, overexpression of miR-486-5p inhibits the development of lung cancer due to the suppression of *GAB2* [157]. Further, decreased level of miRNA-486-5p correlates with KIAA1199 protein overexpression, which in turn results in increased cancer proliferation and poor prognosis [158]. 

Interesting cancer-related features could be attributed also to five miRNA species detected only in exosome-based signatures of lung cancer, namely, let-7f, miR-146, miR-203, miR-106a, and miR-20b. Let-7f belongs to the let-7 (lethal-7) family, which consists of 12 members that regulate cell cycle and cell proliferation by affecting RAS, cyclin A2, CDC34, Aurora A and B kinases, E2F5, CDK8, and HMGA2 [159]. Decreased expression of let-7 is observed in different tumor tissues [160]. Increased expression of let-7f is associated with inhibition of proliferation, migration, and invasion of neoplastic cells, including lung cancer cells, while its decreased expression was observed in metastatic cells [161]. miR-146 is involved in the regulation of inflammation [162]. The overexpression of miR-146 is associated with increased survival and migration of NSCLC cells via suppressing *TRAF6* [163]. Further, increased expression of miR-146 in lung cancer cells lowers the level of claudin-12, which in turn leads to activation of the Wnt/β-catenin and PI3K/AKT/MAPK signaling pathways resulting in the increased viability and migration, as well as resistance to cisplatin and inhibition of apoptosis [164]. Another oncogenic miRNA observed in exosomes of lung cancer patients is miR-106, which increased expression correlates lymph node metastases, drug resistance, and poor prognosis [165]. Increased level of miR-106 decreased expression of *BTG3*, which in turn promotes proliferation and inhibits apoptosis [166]. The expression of miR-20b is also significantly higher in lung cancer cells. miR-20b contributes to the development of NSCLC by inhibiting APC via the canonical Wnt signaling pathway [167]. Moreover, similar to miR-106, miR-20b directly targets *BTG3* [168]. The last miRNA detected in multiple lung cancer signatures is miR-203, which is a putative tumor suppressor. High expression of miR-203 inhibits the proliferation and invasiveness of lung cancer cells through negative regulation of survivin [169]. Moreover, increased expression of miR-203 inhibits *RGS17* oncogene, which results in reduced cell proliferation through the cAMP-PKA-CREB pathway [170]. Furthermore, miR-203 acts as a suppressor of the SRC/Ras/ERK pathway by inhibiting the expression of *SRC* oncogene, resulting in the suppression of proliferation and migration of lung cancer cells [171].

Furthermore, to search systemically for genes regulated by 14 miRNA species that recurred in sEV-based signatures of lung cancer (Figure 2), the miRTarBase database of experimentally validated interactions between miRNA and genes [172] was analyzed. This returned the set of about 600 genes, which functions were analyzed using the FunRich functional enrichment analysis tool [173]. The set comprised of 390 genes associated with lung cancer, including several ones responsible for clinical features of this cancer (e.g., *KRAS*, *EGFR*, *CASP8*, *PIK3CA*, *ERBB2*, *FASLG*, *RB1*, *MYD88*, and *TP53*). Among molecular functions and biological processes associated with this set of genes, several terms potentially involved in cancer development and progression were significantly over-represented, which is summarized in Table 4. Moreover, over-represented biological pathways associated with the most numerous subsets of genes were outweighed by signaling pathways associated with inflammation, immune response, cell growth, cell-to-cell communication, and cancer.

## 6. Conclusions

MicroRNA component of serum/plasma is an attractive source of cancer biomarkers, and several miRNA signatures of lung cancer have been proposed. Though none of them is applied in clinical practice yet, a few are currently tested in prospective clinical trials aimed at validation of their applicability in the early detection of lung cancer and/or diagnosis of the indeterminate pulmonary nodules. Among other potential biomarkers of early lung cancer are exosomes (or rather small extracellular vesicle, sEV) circulating in the blood. Several molecular components of sEV, including proteins, lipids, and non-coding RNAs, have been reported to have different levels in vesicles isolated from lung cancer patients and healthy individuals. The largest number of published reports that address this issue focus on the miRNA component of vesicles. Proposed signatures of exosome miRNA have promising diagnostic value (AUC in the 0.75–0.95 range), yet none of them has been validated in the context of the early detection of lung cancer. These signatures involve a few dozen miRNA species overall, including 14 miRNA (so far) that recurred in different signatures. It is worth noting that all these miRNA species have cancer-related functions and have been associated with lung cancer progression, which further confirms their diagnostic importance. Importantly, a few miRNA species, including known oncomirs miR-17, miR-19, and miR-21, appear in multiple miRNA signatures of lung cancer that are based on both the whole serum/plasma and serum/plasma-derived exosomes. However, one should note, that due to barely standardized methods of sEV isolation, the analysis of exosome miRNA content represents a diagnostic challenge. Therefore, the direct comparison of a diagnostic value of miRNA signature based on the serum/plasma-derived sEV and the whole specimen is desired, which is not available yet.

## Figures and Tables

**Table 1:** Examples of serum/plasma miRNAs as biomarkers of lung cancer.

Biofluid	miRNA Signature	Size of Groups	Diagnostic Value	Reference
Plasma	miR-21, miR-126, miR-210, miR-486	Control: 29 Cases: 29 (Stage I–IV)	AUC = 0.86 SEN = 75% SPE = 85%	[49]
Plasma	miR-21, miR-335	Control: 38 Cases: 36 (Stage I)	AUC = 0.86 SEN = 72% SPE = 81%	[50]
Plasma	miR-21, miR-486	Control: 46 Cases: 54 (Stage I–III)	AUC = 0.90 SEN = 87% SPE = 87%	[51]
Plasma	miR-21, miR-145, miR-155	Control: 92 Cases: 96 (Stage I–IV)	AUC = 0.85 SEN = 69% SPE = 78%	[52]
Plasma	miR-101, miR-106a, miR-126, miR-133a, miR-140-3p, miR-140-5p, miR-142-3p, miR-145, miR-148a, miR-15b, miR-16, miR-17, miR-197, miR-19b, miR-21, miR-221, miR-28-3p, miR-30b, miR-30c, miR-320, miR-451, miR-486-5p, miR-660, and miR-92a (Plasma miR Signature Classifier; MSC)	Control: 870 Cases: 69 (Stage I–III)	SEN = 87% SPE = 81%	[37]
Plasma	miR-182, miR-183, miR-210, miR-126	Control: 40 Cases: 112 (Stage I–III)	AUC = 0.97 SEN = 81% SPE = 100%	[53]
Plasma	miR-145, miR-20a, miR-21, miR-223	Control: 83 Cases: 129 (Stage I–II)	AUC = 0.90 SEN = 82% SPE = 90%	[54]
Plasma	miR-19b, miR-21, miR-221, miR-409, miR-425, miR-584	Control: 124 Cases: 141 (Stage I–IV)	AUC = 0.84 SEN = 73% SPE = 80%	[55]
Serum	miR-92, miR-484, miR-486, miR-328, miR-191, miR-376a, miR-342, miR-331, miR-30c, miR-28, miR-98, miR-17, miR-26b, miR-374, miR-30b, miR-26a, miR-142, miR-103, miR-126, let-7a, let-7d, let-7b, miR-32, miR-133b, miR-566, miR-432, miR-223, miR-29a, miR-148a, miR-142, miR-22, miR-148b, miR-140, miR-139	Control: 69 Cases: 95 (Stage I–IV)	AUC = 0.89 SEN = 71% SPE = 90%	[38]
Serum	miR-15b, miR-27b	Control: 95 Cases: 85 (Stage I–IV)	AUC = 0.98 SEN = 100% SPE = 84%	[56]
Serum	miR-92a-3p, miR-30b-5p, miR-191-5p, miR-484, miR-328-3p, miR-30c-5p, miR-374a-5p, let-7d-5p, miR-331-3p, miR-29a-3p, miR-148a-3p, miR-223-3p, miR-140-5p (miR-Test)	Control: 984 Cases: 48 (Stage I–III)	AUC = 0.85 SEN = 72% SPE = 77%	[48]
Serum	miR-193b, miR-301, miR-141, miR-200b	Control: 45 Cases: 154 (Stage I–III)	AUC = 0.99 SEN = 97% SPE = 96%	[57]
Serum	miR-483, miR-193a, miR-25, miR-214, miR-7	Control: 63 Cases: 63 (Stage I–IV)	AUC = 0.82 SEN = 89% SPE = 68%	[58]
Serum	miR-152, miR-148a, miR-148b, miR-21	Control: 70 Cases: 70 (Stage I–IV)	AUC = 0.97 SEN = 96% SPE = 91%	[59]
Serum	miR-15b, miR-16, miR-20a	Control: 58 Cases: 94 (Stage I–III)	AUC = 0.93 SEN = 86% SPE = 91%	[60]
Serum	miR-429, miR-205, miR-200b, miR-203, miR-12, miR-34b	Control: 74 Cases: 138 (Stage I–IV)	AUC = 0.89 SEN = 88% SPE = 71%	[61]
Serum	miR-141, miR-193b, miR200b, miR-301	Control: 185 Cases: 213 (Stage I–IV)	AUC = 0.92 SEN = 91% SPE = 78%	[62]
Serum	miR-1268b, miR-6075	Control: 2178 Cases: 1566 (Stage I–IV)	AUC = 0.99 SEN = 99% SPE = 99%	[63]

AUC—Area Under the Receiver Operating Characteristic (ROC) Curve; SEN—Sensitivity; SPE—Specificity.

**Table 2:** Potential exosome biomarkers of lung cancer.

Biofluid/EV Isolation	Size of Groups	Proposed Biomarker	Analytic. Method	Diagnostic Value	Reference
Serum/UC TEM, NTA, WB	Control: 46 Cases: 125 (Stage I–IV)	AHSG, ECM1 proteins	MS	AUC = 0.80 SEN = 54% SPE = 89%	[104]
Serum/IMA	Control: 10 Cases: 26 (Stage III–IV)	CD91	MS	AUC = 0.72 SEN = 60% SPE = 89%	[105]
Plasma/UC TEM, NTA, WB	Control: 15 Cases: 13 (Stage I–II)	SRGN, TPM3, THBS1, HUWE1 proteins	MS	AUC = 0.90 SEN = 81% SPE = 82%	[106]
Serum/UC TEM, NTA, WB	Control: 90 Cases: 183 (Stage I–IV)	LPS-binding protein (LBP)	ELISA	AUC = 0.71 SEN = 65% SPE = 76%	[107]
Plasma/EV array	Control: 150 Cases: 431 (Stage I–IV)	CD151, Tspan8, NYESO1, HER2, CD171, EGFRvIII SFTPD, Flotilin1, CD142, Mucin16	EV array	AUC = 0.74 SEN = 71% SPE = 69%	[103]
Serum/PRE TEA, NTA	Control: 150 Cases: 150 (Stage I–IV)	lncRNA (TBILA)	qPCR	AUC = 0.78 SEN = 65% SPE = 81%	[108]
Serum/PRE TEA, NTA	Control: 150 Cases: 150 (Stage I–IV)	lncRNA (AGAP2-AS1)	qPCR	AUC = 0.73 SEN = 67% SPE = 73%	[108]
Serum/PRE TEM, NTA, WB	Control: 64 Cases: 72 (Stage I–IV)	lncRNA (DLX6-AS1)	qPCR	AUC = 0.81 SEN = 78% SPE = 86%	[109]
Serum/PRE TEM, NTA, WB	Control: 30 Cases: 77 (Stage I–IV)	lncRNA (MALAT-1)	qPCR	AUC = 0.70 SEN = 60% SPE = 81%	[85]
Serum/PRE TEM, NTA, WB	Control: 40 Cases: 64 (Stage I–IV)	lncRNA (GAS5)	qPCR	AUC = 0.86 SEN = 86% SPE = 70%	[110]
Serum/PRE WB	Control: 30 Cases: 120 (Stage I–IV)	circular RNA (circRNA-002178)	qPCR	AUC = 0.99 SEN = 99% SPE = 100%	[111]
Plasma/UC	Control: 39 Cases: 44 (Stage I–II)	PC(32:0), PC(34:2), PC(36:1)/(36:2)/(36:3), PC(38:3)/(38:5)/(38:6), LPC(12:0), LPC(16:0), SM(34:1), SM(42:2), TG(52:5), TG(54:6), CE(20:4)	MS	AUC = 0.85 SEN = 77% SPE = 72%	[112]

sEV’s isolation and characterization methods: UC—Ultracentrifugation; PRE—Precipitation; IMA—Immunoaffinity; TEM—Transmission Electron Microscopy; NTA—Nanoparticle Tracking Analysis; WB—Western Blot; MS—mass spectrometry; qPCR—quantitative real-time PCR; AUC—Area Under the ROC Curve; SEN—Sensitivity; SPE—Specificity.

**Table 3:** Potential sEV miRNA biomarkers of lung cancer.

Biofluid/EV Isolation	miRNA Signature	Size of Groups	Diagnostic Value	Reference
Plasma/PRE	miR-378a, miR-379, miR-139-5p, miR-200b-5p	Control: 25 Cases: 80 (Stage I)	AUC = 0.91 SEN = 98% SPE = 72%	[113]
Plasma/PRE WB, TEM	miR-30b, miR-30c, miR-103, miR-122, miR-195, miR-203, miR-221, miR-222	Control: 6 Cases: 12 (Stage -)	-	[114]
Plasma/PRE	miR-19-3p, miR-21-5p, miR-221-3p	Control: 14 Cases: 18 (Stage I–IV)	-	[55]
Plasma/PRE WB, NTA, TEM	miR-23b-3p, miR-10b-5p, miR-21-5p	Control: 10 Cases: 10 (Stage I–IV)	AUC = 0.91 SEN = 82% SPE = 85%	[115]
Plasma/PRE WB, NTA, TEM	miR-451a, miR-194-5p, miR-486-5p	Control: 149 Cases: 434 (Stage I–IV)	AUC = 0.97 SEN = 95% SPE = 71%	[36]
Plasma/PRE WB, NTA, TEM	miR-185-5p, miR-32-5p, miR-140-3p, let-7f-5p	Control: 20 Cases:79 (Stage I–III)	AUC = 0.91 SEN = 59% SPE = 100%	[116]
Plasma/SEC + IMA	miR-17-3p, miR-21, miR-106a, miR-146, miR-155, miR-191, miR-192, miR-203, miR-205, miR-210, miR-212, miR-214	Control: 8 Cases: 28 (Stage I–IV)	-	[117]
Plasma/IMA	let-7f, miR-20b, miR-30e-3p, miR-223, miR-301	Control: 48 Cases:78 (Stage I–IV)	-	[118]
Plasma/UC + IMA WB, NTA	let-7b-5p, let-7e-5p, miR-24-5p, miR-21-5p	Control: 13 Cases: 47 (Stage I)	AUC = 0.90 SEN = 80% SPE = 92%	[119]
Plasma/UC TEM	miR-21, miR-4257	Control: 30 Cases: 195 (Stage I-III)	-	[120]
Plasma/SEC	miR-411-5p	Control: 7 Cases: 19 (Stage -)	-	[121]
Serum/PRE	miR-451a, miR-486-5p, miR-363-3p, miR-660-5p, miR-15b-5p, miR-25-3p, miR-16-2-3p	Control: 10 Cases: 20 (Stage I–IV)	AUC = 0.98 SEN = 100% SPE = 90%	[122]
Serum/PRE WB, NTA, TEM	miR-17-5p	Control: 137 Cases: 172 (Stage I–III)	AUC = 0.74 SEN = 67% SPE = 77%	[123]
Serum/PRE WB, NTA, TEM	miR-146a-5p, miR-486-5p	Control: 80 Cases: 48 (Stage I–II)	AUC = 0.90 SEN = 83% SPE = 90%	[124]
Serum/PRE	miR-216b	Control: 60 Cases: 105 (Stage I–IV)	AUC = 0.84 SEN = 87% SPE = 75%	[125]
Serum/PRE WB, TEM	miR-106b	Control: 72 Cases: 72 (Stage I–IV)	-	[126]
Serum/PRE	106a-5p, miR-20a-5p, miR-93-5p	Control: 36 Cases: 34 (Stage I–III)	AUC = 0.83	[127]
Serum/PRE WB, NTA, TEM	miR-210-5p, miR-1269a, miR-205-5p, miR-9-3p	Control: 150 Cases: 148 (Stage I–III)	AUC = 0.74 SEN = 81% SPE = 61%	[128]
Serum/PRE WB, NTA, TEM	miR-1290	Control: 40 Cases: 70 (Stage I–IV)	AUC = 0.94 SEN = 80% SPE = 97%	[129]
Serum/PRE	miR-378	Control: 60 Cases: 103 (Stage I–IV)	AUC = 0.84 SEN = 78% SPE = 82%	[130]
Serum/PRE WB, TEM	miR-7977, miR-98-3p	Control: 65 Cases: 65 (Stage I–IV)	AUC = 0.82 SEN = 81% SPE = 75%	[131]
Serum/UC WB, NTA, TEM	miR-126	Control: 31 Cases: 45 (Stage I–III)	AUC = 0.84 SEN = 90% SPE = 86%	[132]
Serum/UC WB, NTA, TEM	miR-21-5p, miR-126-3p, miR-140-5p	Control: 16 Cases: 23 (Stage I–IV)	-	[133]
Serum/UC WB, NTA, TEM	miR-620	Control: 231 Cases: 235 (Stage I–IV)	AUC = 0.71 SEN = 63% SPE = 68%	[134]
Serum/UC WB, NTA, TEM	miR-5684, miR-125b-5p	Control: 312 Cases: 330 (Stage I–IV)	AUC = 0.74 SEN = 81% SPE = 61%	[135]
Serum/UC WB, NTA, TEM	miR-20b-5p, miR-3187-5p	Control: 30 Cases: 380 (Stage 0–I)	AUC = 0.84	[136]

sEV’s isolation and characterization methods: UC—Ultracentrifugation; PRE—Precipitation; IMA—Immunoaffinity; SEC—Size Exclusion Chromatography; TEM—Transmission Electron Microscopy; NTA—Nanoparticle Tracking Analysis; WB—Western Blot; AUC—Area Under the ROC Curve; SEN—Sensitivity; SPE—Specificity.

**Table 4:** Functions associated with genes regulated by exosome miRNAs common in lung cancer signatures.

**Molecular Function**	**No. of Genes**	**Fold Enrichment**	**FDR**
Transcription factor activity	72	2.64	<0.00001
Receptor activity	32	2.73	0.00003
Protein serine/threonine kinase activity	28	2.87	0.00005
Transmembrane receptor protein tyrosine kinase activity	11	6.06	0.00008
Receptor signaling complex scaffold activity	28	2.68	0.00010
Receptor binding	16	3.83	0.00016
Protein-tyrosine kinase activity	8	6.50	0.00077
Transcription regulator activity	47	1.74	0.00451
GTPase activity	18	2.50	0.00868
Kinase regulator activity	5	6.71	0.01639
**Biological Process**	**No. of Genes**	**Fold Enrichment**	**FDR**
Signal transduction	240	1.88	<0.00001
Cell communication	223	1.85	<0.00001
Regulation of nucleotide and nucleic acid metabolism	144	1.57	<0.00001
Apoptosis	26	3.64	<0.00001
Regulation of cell growth	5	7.35	0.01662

No. of genes—number of genes connected to specific term among 600 genes in the whole set; FDR—corrected p-value of the hypergeometric test for the significance of over-representation.

## Data Availability

Not applicable.
